# Material Engineering Strategies for Efficient Hydrogen Evolution Reaction Catalysts

**DOI:** 10.1002/smtd.202400158

**Published:** 2024-05-15

**Authors:** Yue Luo, Yulong Zhang, Jiayi Zhu, Xingpeng Tian, Gang Liu, Zhiming Feng, Liwen Pan, Xinhua Liu, Ning Han, Rui Tan

**Affiliations:** ^1^ School of Resources Environment and Materials Guangxi University Nanning 530004 China; ^2^ College of Mechatronical and Electrical Engineering Hebei Agricultrual Univesity Baoding 07001 China; ^3^ Warwick Electrochemical Engineering WMG University of Warwick Coventry CV4 7AL UK; ^4^ IDTECH (Suzhou) Co. Ltd. Suzhou 215217 China; ^5^ Department of Chemical Engineering Imperial College London London SW7 2AZ UK; ^6^ Education Department of Guangxi Zhuang Autonomous Region Key Laboratory of High Performance Structural Materials and Thermo‐surface Processing (Guangxi University) Nanning 530004 China; ^7^ School of Transportation Science and Engineering Beihang University Beijing 100191 China; ^8^ Department of Materials Engineering KU Leuven Kasteelpark Arenberg 44, bus 2450 Heverlee B‐3001 Belgium; ^9^ Department of Chemcial Engineering Swansea University Swansea SA1 8EN United Kingdom

**Keywords:** catalytic materials, design principles, hydrogen evolution reaction, material engineering strategies, noble metal‐free catalysts

## Abstract

Water electrolysis, a key enabler of hydrogen energy production, presents significant potential as a strategy for achieving net‐zero emissions. However, the widespread deployment of water electrolysis is currently limited by the high‐cost and scarce noble metal electrocatalysts in hydrogen evolution reaction (HER). Given this challenge, design and synthesis of cost‐effective and high‐performance alternative catalysts have become a research focus, which necessitates insightful understandings of HER fundamentals and material engineering strategies. Distinct from typical reviews that concentrate only on the summary of recent catalyst materials, this review article shifts focus to material engineering strategies for developing efficient HER catalysts. In‐depth analysis of key material design approaches for HER catalysts, such as doping, vacancy defect creation, phase engineering, and metal‐support engineering, are illustrated along with typical research cases. A special emphasis is placed on designing noble metal‐free catalysts with a brief discussion on recent advancements in electrocatalytic water‐splitting technology. The article also delves into important descriptors, reliable evaluation parameters and characterization techniques, aiming to link the fundamental mechanisms of HER with its catalytic performance. In conclusion, it explores future trends in HER catalysts by integrating theoretical, experimental and industrial perspectives, while acknowledging the challenges that remain.

## Graphical abstract

This perspective article focuses on the development of cost‐effective and high‐performance noble metal‐free catalysts for hydrogen evolution reaction (HER) in water electrolysis, aiming to enable widespread deployment of this technology for achieving net‐zero emissions. It highlights material engineering strategies, including doping, vacancy defect creation, phase engineering, and metal‐support engineering. Finally, an outlook on non‐noble metal catalysts is presented from three perspectives: theoretical, experimental, and industrialization.

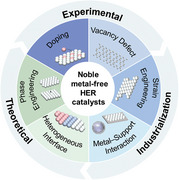

## 1 Overview

Among various carbon‐free energy sources, hydrogen has been regarded as a promising energy carrier due to its green and high‐efficiency properties, holding the potential as a high‐energy resource for energy storage and transportation.^[^ ^1^ ^]^ Hydrogen can be generally produced from fossil fuels, specifically natural gas, and renewable energy sources such as water, wind and solar power. Major industrial methods for large‐scale hydrogen production include steam methane reforming, coal gasification and water electrolysis.^[^ ^2^ ^]^ The first two processes constitute 95% of industrial hydrogen production,^[^ ^3^ ^]^ but their heavy reliance on the use of fossil fuels raises significant energy and environmental concerns. Conversely, water electrolysis is a more promising technology for carbon‐free hydrogen production, as it avoids the use of non‐renewable fossil fuels and does not produce harmful emissions. The economic costs of electrolyzed water constrain its potential for significant market growth in industrial hydrogen production. Urgent attention is required to address this issue, focusing on technological advancements and material innovations to change the current circumstances.

Water electrolysis is an energy‐consuming reaction that requires energy input to overcome the obstacles in the electrochemical process to produce gas. Hydrogen evolution reaction (HER) is the key reaction process involved in water electrolysis, requiring appropriate catalysts to overcome the overpotential generated during hydrogen production.^[^ ^4^ ^]^ Therefore, the primary objective of enabling efficient HER is to develop catalysts that are active and stable so as to minimize the reaction overpotential. Research on HER mechanism has been ongoing and has gained momentum. Over the past few decades, significant progress has been made through the accumulation of experimental knowledge, leading to a fundamental understanding of HER in its simplest form. Theoretical models based on density‐functional theory (DFT) have emerged as crucial tools for exploring the catalytic process of HER. DFT offers in‐depth insights into the atomic‐level catalytic mechanism, enhancing our comprehensive understanding of the electrocatalytic process.^[^ ^5^ ^]^ Descriptors facilitate the integration of theoretical and experimental results, enable qualitative assessment of catalysts and guide material design.^[^ ^6^ ^]^

Most commercial HER electrocatalysts are based on platinum‐based noble metals and their derivatives.^[^ ^7^ ^]^ Although noble metal‐based catalysts like platinum (Pt) exhibit low overpotential in electrocatalysis, their high cost impedes large‐scale application. Consequently, researchers are focusing on cost‐effective, noble metal‐free catalysts for hydrogen evolution electrocatalysis.^[^ ^8^, ^9^, ^10^, ^11^ ^]^ Extensive studies have identified the essential attributes of excellent catalysts, including high intrinsic activity, efficient charge transfer, and a high density of active sites.^[^ ^12^, ^13^ ^]^ These characteristics are fundamental in guiding catalyst design. Based on them, relevant strategies that enhance both the intrinsic activity and external modulation of catalysts are being developed. The exploration of controllable optimization strategies is crucial for significantly enhancing energy and power density, catalytic activity, efficiency and durability, thereby meeting the future demands for electrochemical energy conversion and storage.

Certainly, vast review articles concentrate only on the summary of reported materials and their electrochemical performance. Distinct from these typical articles, this work shifts focus to material engineering approaches, e.g., doping, phase and strain engineering, metal‐support interaction, and propose catalyst design through a top‐down design method. Catalytic materials can be engineered for the following purposes: 1) enhance active‐site density; 2) decrease interfacial transfer resistance and improve conductivity; 3) enhance reaction kinetics and reduce hydrogen adsorption Gibbs free energy and 4) establish stable structure. The article concludes with a forward‐looking view on future HER catalysts and advanced techniques.

## 2 Mechanistic and Theoretical of HER

### 2.1 HER Mechanism

Prototypical catalytic hydrogen production usually occurs in an electrolytic cell that composes of aqueous electrolyte, cathodes and anodes. With supplied current, this electrolytic cell involves two seemingly simple reactions, HER and oxygen evolution reaction (OER). Catalysts are necessarily placed onto cathodes and anodes to enable HER and OER, respectively, of which electrochemical processes vary in the aqueous electrolyte with varied pH. The overall reactions are provided below.

(1) $$\mathrm{Total}\;\mathrm{reaction}:\;H_{2}O\to H_{2}+\frac{1}{2}O_{2}$$

In acidic solutions:

(2) $$\mathrm{Cathode}:\;2H^{+}+\;2e^{-}\to \;H_{2}$$

(3) $$\mathrm{Anode}:\;H_{2}O\;\to \;H^{+}+\;\frac{1}{2}O_{2}+\;2e^{-}$$

In neutral and alkaline solution:

(4) $$\mathrm{Cathode}:\;2H_{2}O+\;2e^{-}\to \;H_{2}+2OH^{-}$$

(5) $$\mathrm{Anode}:\;2OH^{-}\to \;H_{2}O+\;\frac{1}{2}O_{2}+2e^{-}$$

Theoretical thermodynamic voltage of electrolyzed water is 1.23 V (101.3 kPa, 25 °C) based on the Nernst equation. However, overpotentials at the anode (η_*a*_) and cathode (η_*c*_) and ohmic polarization (η_*other*_) undesirably enlarge the water electrolysis voltage (*E_P_*) in practical scenarios.^[^ ^3^ ^]^ The below equation demonstrates that enhancing the water electrolysis efficiency necessitates the reduction of overpotentials.

(6) $$E_{P}=\;1.23V+\;\eta_{a}+\;\eta_{c}+\;\eta_{\mathrm{other}}\;$$

**Figure 1:**
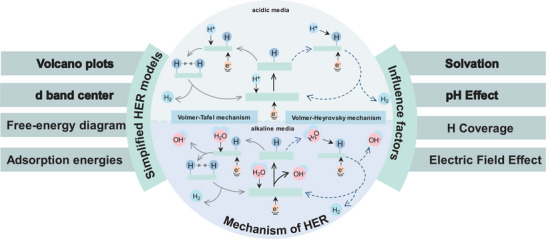
HER mechanisms in acidic and alkaline conditions, simple theoretical models and influencing factors. (More important models and influences are indicated in bold).

HER is a two‐electron transfer yet multi‐step process where “adsorption–complexation–desorption” occurs on the surface of the catalyst. A wealth of data accumulation and experimental analysis has significantly enhanced our knowledge of the kinetics and mechanisms of the HER. The well‐established reaction mechanisms and theoretical models in HER are concisely summarized in **Figure** **1**.

In acidic electrolyte, the first step is the Volmer process where hydrated hydrogen ions (H_3_O^+^) diffuse and adsorb at the active reaction sites (*) on the catalyst surface, followed by their combination with electrons (Equation 7), leading to the formation of hydrogen adsorption intermediates (H^*^) (Figure 1a). Subsequently, these intermediates can undergo two types of complexations: the first is known as the **Heyrovsky reaction**, in which H^*^ combines with H_3_O^+^ in the solution and electrons and results in desorption and the production of H_2_ (Equation 8). The second process is referred to as the **Tafel reaction**, wherein two H^*^ intermediates combine and subsequently desorb to generate H_2_ (Equation 9).

(7) $$\mathrm{Volmer}\;\mathrm{reaction}:\;\;H_{3}O^{+}+\;*\;+\;e^{-}\to H^{*}+\;H_{2}O$$

(8) $$\mathrm{Heyrovsky}\;\mathrm{reaction}:\;\;H_{3}O^{+}+H^{*}+\;e^{-}\to H_{2}+\;H_{2}O+\;*$$

(9) $$\mathrm{Tafel}\;\mathrm{reaction}:\;\;H^{*}+H^{*}\to \;H_{2}+2*$$

Similarly, two distinct HER reaction pathways, the Volmer–Heyrovsky reaction (Equations 10 and 11) and the Volmer–Tafel reaction (Equations 10 and 12), are involved within alkaline electrolyte (Figure 1b). Herein, H_2_O functions as the sole proton donor in the environment and undergoes dissociation to generate H^+^ and OH^−^ ions. Subsequently, H^*^ form at the active reaction site (*).

(10) $$\mathrm{Volmer}\;\mathrm{reaction}:\;\;H_{2}O+\;*\;+\;e^{-}\to H^{*}+\;OH^{-}$$

(11) $$\mathrm{Heyrovsky}\;\mathrm{reaction}:\;\;H_{2}O+H^{*}+\;e^{-}\to H_{2}+OH^{-}+\;*$$

(12) $$\mathrm{Tafel}\;\mathrm{reaction}:\;\;\;H^{*}+H^{*}\to \;H_{2}+2*$$

Prior to the formation of adsorbed H^*^ species in alkaline electrolyte, efficient catalysts are required to break H─O─H bonds of water. As a result, the activity in alkaline media is significantly lower by 2–3 orders of magnitude in comparison to acidic solutions.^[^ ^14^, ^15^ ^]^ In addition to focusing on reaction pathway of the hydrogen ions, it is important to consider the activation energy required for water dissociation and the resulting effects of OH^−^ production. Consequently, the reaction mechanism of HER in alkaline conditions is more complex.^[^ ^16^ ^]^

### 2.2 Main Descriptor for HER Performance

To explore the mechanism and operating principles of the HER at the atom scale, theoretical calculations, especially DFT, are employed to derive HER descriptors. DFT is pivotal in these theoretical calculations and contributes to two aspects. First, it allows for the quantitative determination of adsorption energy and free energy through energy calculations, enabling an analysis of their interrelationship. Second, DFT utilizes band structure analysis to derive the well‐established band theory. Understanding the structure‐activity relationship of HER catalysts through these descriptors enables a thorough assessment of their catalytic activity.^[^ ^5^ ^]^

**Figure 2:**
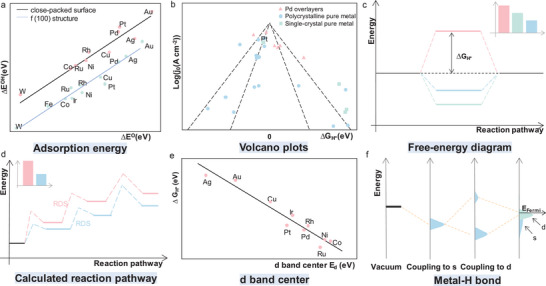
Theoretical models related to HER. a) Adsorption energies of intermediates. b) Volcano plot of *j*_0_ as a function of $\Delta G_{H^{*}}$. c) Free‐energy diagram at equilibrium potential. d) Calculated reaction pathways for the electrocatalytic process. e) Relations between d band center *E*_d_ with hydrogen binding strength $\Delta G_{H^{*}}$ f) Schematic illustration of the metal─H bond.

The computational hydrogen electrode (CHE) is commonly used to simulate various electrocatalytic reactions due to its thermodynamic rigor and computational efficiency.^[^ ^17^ ^]^ In the case of HER, this simplified model can be used to calculate the hydrogen adsorption energy using gaseous H_2_ as reference state (**Figure** **2**a):^[^ ^18^ ^]^

(13) $$\Delta E_{H^{*}}=\;E-E_{C}-\frac{1}{2}E_{H_{2}}$$

where *E* refer to the total energy of catalyst hydrogen adsorption,*E_C_* refer to the catalyst energy, and $E_{H_{2}}$ refer to the energy of gaseous H_2_. Subsequently, the Gibbs energy of hydrogen adsorption ($\Delta G_{H^{*}}$) can be calculated according to the Equation (14).

(14) $$\Delta G_{H^{*}}=\;\Delta E_{H^{*}}\;+\;\Delta E_{\mathrm{ZPE}}-\;T\Delta S+\Delta G_{\mathrm{pH}}$$

where Δ*E_ZPE_* is the change of zero‐point energy. Δ*S* represents the change in entropy when gaseous H_2_ transforms into adsorbed H* under standard conditions, which is roughly half the entropy of gaseous H_2_. The pH effect on the thermodynamics is described by Δ *G_pH_* =  *pH* × *k_B_T*ln 10, where *k_B_* is the Boltzmann constant. CHE model has shown considerable success in predicting the activity and intermediates within HER processes. However, CHE model primarily focuses on thermodynamics but tends to overlook the factors related to kinetics, solvation, and capacitance.

∆G_H*_ acts as a descriptor for the catalytic activity of a reaction. Incorporating the steps of the HER and the reaction potential barrier, a standard free energy diagram is constructed (Figure 2c). The Sabatier principle that effective reactions require optimal interactions between the catalyst surface and reaction intermediates — neither too strong or too weak.^[^ ^19^ ^]^ According to the above principle, a $\Delta G_{H^{*}}$ value greater than zero indicates weak hydrogen binding ability, leading to low catalytic activity. Conversely, when $\Delta G_{H^{*}}$< 0, it signifies a strong hydrogen binding ability and promotes proton‐coupled electron transfer (PCET).^[^ ^20^ ^]^ Therefore, an ideal HER catalyst exhibits a $\Delta G_{H^{*}}$ value close to zero.^[^ ^21^ ^]^ This concept led to the development of “volcano plot”.^[^ ^22^ ^]^ Plotting the $\;\Delta G_{H^{*}}$ values from DFT calculations of various metal‐based catalysts against the exchange current densities(*j*_0_) from experimental tests, the volcano plot aids in assessing intrinsic catalytic activity and guiding catalyst performance optimization (Figure 2b).^[^ ^23^, ^24^ ^]^

The kinetics of the HER are crucial in the design and optimization of HER catalyst performance. The overall reaction rate is governed by the rate‐determining step (RDS) which could be one of the various elementary reactions like Volmer, Tafel, or Heyrovsky in the HER process. The energy barrier (Δ*G_RDS_*) for different RDSs exhibits variability with a lower barrier indicating faster reaction kinetics (Figure 2d). By strategically influencing the RDS of certain pathways, the activity of HER catalysts can be enhanced. Currently, the Tafel slope is used to understand the HER reaction mechanism and identify RDS. The kinetic expressions and corresponding Tafel slopes are summarized in **Table** **1**.

**Table 1:** Kinetic expressions and Tafel slopes for HER with different RDS.

Mechanism	RDS	Kinetic expression ^a)^	Tafel slope ^b)^
Volmer–Tafel	Volmer	$i\;=\;2i_{0}[-e^{\frac{-\alpha F}{\mathrm{RT}}\eta }+e^{\frac{\beta F}{\mathrm{RT}}\eta }]$	$\frac{2.303\mathrm{RT}}{\alpha F}$
	Tafel	$\eta \;=\frac{\mathrm{RT}}{2F}\;\ln\left(1+\frac{i}{\mathrm{iT}}\right)$	$\frac{2.303\mathrm{RT}}{2F}$
Volmer–Heyrovsky	Volmer	$i\;=\;2i_{0}[-e^{\frac{-\alpha F}{\mathrm{RT}}\eta }+e^{\frac{(1+\beta )F}{\mathrm{RT}}\eta }]$	$\frac{2.303\mathrm{RT}}{\alpha F}$
	Heyrovsky	$i\;=\;2i_{0}[-e^{\frac{-(1+\alpha )F}{\mathrm{RT}}\eta }+e^{\frac{\beta F}{\mathrm{RT}}\eta }]$	$\frac{2.303\mathrm{RT}}{(1+\alpha )F}$

^a)^ i_0_ is exchange current, η is overpotential;

^b)^ α is the electron transfer coefficient, α  =  0.5.

While the Butler–Volmer equation sheds light on the kinetics and reaction mechanism of electrode reactions, it falls short in explaining the influence of factors such as catalyst structure, solvent, and electrode material on the kinetics.^[^ ^25^ ^]^ To fully grasp these aspects, it is essential to gain insight into the microscopic perspective of electron transfer between substances. To elucidate the correlation between the rate of charge transfer, reorganization energy, and Gibbs free energy change, it is beneficial to integrate Marcus theory. It provides a more detailed understanding of microscopic charge transfer processes, particularly PCET on the electrode surface. In the HER, the reaction involves two proton transfers and two electron transfers: heterogeneous HER forms intermediates through stepwise electron transfer‐proton transfer (ET‐PT), while homogeneous HER may proceed either through cooperative proton coupled electron transfer or stepwise ET‐PT (or PT‐ET).^[^ ^6^, ^26^ ^]^ Currently, to gain a more comprehensive understanding of these intricate kinetic processes, a combination of extensive experimental work and theoretical calculations is greatly needed.^[^ ^27^ ^]^

The d‐band model, which is a valuable tool for predicting the chemical adsorption behavior of diverse adsorbates on metal surfaces, this extensively utilized model enables theoretical descriptions of the reactivity and adsorption energy of reactions and intermediates on metal, metal alloy, and oxide surfaces.^[^ ^28^, ^29^ ^]^ Its core principle is that the binding energy between adsorbates and metal surfaces is primarily influenced by the electronic structure. As the s‐band bonding process undergoes minimal energy changes, electrons in the d orbitals of transition metals become the principal contributors to chemical bonding. Specifically, hydrogen interacts with the electrons in the metal d orbitals of the catalyst, forming chemical bonds. Quantitative calculation of the interaction between the d‐band center of transition metals and adsorbates enable the discovery of highly active novel catalysts (Figure 2e).^[^ ^30^ ^]^ The adsorbate hydrogen binds to the transition metal surface, occupying both bonding and antibonding orbitals. The efficacy of the catalyst depends on the metal–H interaction and the degree of orbital occupancy (Figure 2f). The strengthening or weakening of the metal─H bond is determined by the shift of the d‐band center in relation to the Fermi level.^[^ ^21^, ^29^, ^31^ ^]^

Besides the above descriptors, specific descriptors have been developed for certain types of catalytic materials. It is important to recognize that each descriptor has its own limitations and is not universally applicable to all catalysts. For instance, regarding transition metals, the lowest unoccupied state of disulfides serve as a crucial descriptor for surface activity.^[^ ^32^ ^]^ In the case of non‐metal carbon materials, band theory acts as an activity descriptor that can predict the catalytic activity.^[^ ^33^, ^34^ ^]^ Additionally, the generalized coordination number descriptor predicts the optimal geometric shape of catalytic sites, taking into account the interactions among nearest and next‐nearest neighbor elements.^[^ ^35^, ^36^ ^]^

### 2.3 Influencing Factors of HER

The descriptors mentioned earlier are extensively utilized to validate the HER performance of catalysts and to provide a mechanistic explanation. However, under practical conditions, it is different to obtaining accurate catalytic activity data or elucidating the working principles only based on the simplified HER model.^[^ ^37^ ^]^ Other influential factors, including solvation, pH effects, hydrogen coverage and electric field effects should be considered.

The interaction between reaction intermediates and the surrounding solvent is crucial in the HER. Of particular significance is the electrochemical double layer, capable of withstanding potential changes of several volts within a thin layer of 3–5 Å. The behavior of solvent molecules within such a strong electric field can significantly impact the principles of electrocatalysis.^[^ ^38^ ^]^ However, both experimental and computational detection of solvation remains challenging, necessitating more refined models for theoretical calculations. Currently, theoretical calculations often use two types of models: implicit and explicit.^[^ ^39^ ^]^ Explicit solvent models are particularly used for examining the influence of hydrogen bonding on reaction processes and the role of solvents in proton transfer processes. They are, therefore, frequently employed to investigate HER mechanisms, despite their high computational cost. Implicit solvent models, in contrast, are better suited for understanding the effects of defects and dispersion on the interactions between solute and solvent.^[^ ^40^ ^]^ Each model offers unique insights, highlighting the need for continued development in this area to fully comprehend solvation effects in electrocatalysis.

In the experimental study, it is commonly observed that the conversion of H_3_O^+^ to H_2_O can vary by approximately two orders of magnitude with changes in pH, significantly affecting the reaction rate. However, there is no consensus yet on the explanation of this pH effect. Currently, three main hypothesis are being considered.^[^ ^41^ ^]^

1. The first explanation resolves around the fluctuations in proton donors, which vary with the pH of the electrolyte.^[^ ^42^ ^]^ This variation could influence the availability of protons necessary for HER.
2. The second theory suggests that the unique structure of hydrogen bonding networks within the electrochemical double layer might governs the pH‐dependent kinetics of HER.^[^ ^43^ ^]^ The way these networks are organized could affect the reaction rate.
3. The third hypothesis points to the role of interface solvent dynamics and water recombination effects, which are believed to depend on the pH values.^[^ ^44^, ^45^ ^]^ This involves how the behavior of solvent molecules at the interface influences the reaction kinetics.

Water electrolysis releases H^+^ which binds to the catalyst surface and undergoes hydrogen recombination to produce molecular hydrogen. The slow hydrolysis dissociation reaction in neutral/alkaline media results in low H coverage on the HER catalyst surface, which inhibits HER catalysis.^[^ ^46^ ^]^ In addition, in electrochemical processes sensitive to surface interactions, hydrogen coverage and competing adsorption between active species cannot be neglected, which can significantly affect reaction efficiency.^[^ ^47^ ^]^ Furthermore, low‐dimensional semiconductor catalysts for the HER, such as transition metal dichalcogenides, are particularly responsive to external electric fields. In alkaline environments, the directional movement of interface water is altered by local electric fields. This directional movement of interface water, dictated by the electric fields, plays a key role in affecting the catalytic activity of these materials.^[^ ^48^ ^]^

## 3 Experimental Methods and Characterization Techniques

**Figure 3:**
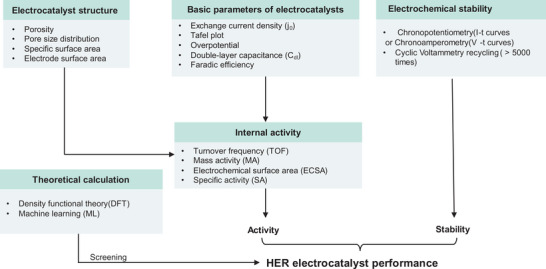
Correlation of fundament metrics, materials attributes/properties, and performance. The use of theoretical calculations can help to identify materials suitable for use as HER catalysts. Data obtained from testing and characterization can provide a crucial basis for evaluating the potential of these materials to exhibit excellent HER catalytic effects.

Electrochemical testing methods and characterization techniques are important for catalyst performance evaluation, mechanism investigation and material optimization. **Figure** **3** correlates the evaluation protocols with important parameters for noble metal‐free catalysts. By comprehensively analyzing these indicators, comprehensive evaluation approaches and optimization results can be obtained and used to guide catalyst designs and applications.

### 3.1 Electrochemical Parameters

#### 3.1.1 Electrochemical Activity

Cyclic voltammetry (CV), Linear sweep voltammetry (LSV) and Electrochemical impedance spectroscopy (EIS) are three commonly used protocols to evaluate the electrochemical performance of catalysts by specific metrics, including overpotential, Tafel slope, exchange current density, and double‐layer capacitance (C_dl_).

**Overpotential** refers to the disparity between the practical potential and the equilibrium potential with an applied current,^[^ ^49^ ^]^ partially induced by the ohmic resistance of electrolyte and physical contact among accessories,^[^ ^50^ ^]^ which necessitates the compensation of iR where R represents the ohmic resistance. The **Tafel slope** obtained from plotting overpotential against the logarithm of exchange current density, serves as a key evaluation parameter for electrochemical activity. A smaller Tafel slope suggests that the catalyst can produce a certain output of current at a lower potential, indicating superior electrocatalytic activity.

While the parameters mentioned above can evaluate the overall catalytic performance of materials, they may not effectively measure the intrinsic properties of catalysts. To address this, four main indicators are suggested, including **turnover frequency** (TOF), **specific activity** (SA), **electrochemical surface area** (ECSA), and **mass activity** (MA). TOF refers to the rate of reactions that occur per active site per unit of time, considering factors like pressure, temperature, reactant ratio and reaction progress.^[^ ^51^ ^]^ SA calculates the current density of the catalyst per unit electrochemical active surface area and unit catalyst mass. Combining TOF with SA allows for comparing the intrinsic activity of various HER catalysts and an approximate assessment of the density of effective catalytic sites. ECSA quantifies the effective surface area of the catalyst under electrochemical conditions, usually estimated through measurement. It is used to evaluate the electrochemical activity and surface reactivity of catalysts, indicating the ability of catalyst for exchanging electrons and ions.^[^ ^52^ ^]^ Meanwhile, MA measures the catalytic activity per unit mass of the catalyst, considering both the total activity and mass of the catalyst, which is useful for evaluating the overall performance and efficiency. However, quantifying the number of active sites poses a challenge due to the dynamic nature of catalyst surfaces and the reconstruction they undergo during the reaction process.^[^ ^53^ ^]^

#### 3.1.2 Electrochemical Stability

While research on the activity of HER electrocatalysts has been extensive, stability is another crucial aspect for practical applications, often seen as a key factor for potential commercialization. There are primarily two traditional methods for catalyst stability testing, one by chronopotentiometry or chronoamperometry; the other by CV that usually cycles for above 5000 times to evaluate the practical catalytic performance. In addition to these approaches, in situ characterization techniques are used to precisely detect catalyst dissolution during the reaction, thereby offering a more durable evaluation of stability,^[^ ^54^ ^]^ e.g., differential electrochemical mass spectrometry and flow cell‐inductively coupled plasma‐mass spectrometry.

In addition to comprehending the electrochemical stability testing of catalysts, it is crucial to make comprehensive assessments. These assessments should consider factors such as the catalyst's structural changes before and after testing and the impact of catalyst loading. Following the test, surface remodeling, active site poisoning, and demetallization may occur during the reaction.^[^ ^54^, ^55^, ^56^ ^]^ At the same current density, a balance between loading and catalyst stability must be achieved. While increasing the catalyst loading to some extent can enhance catalytic activity, it does not necessarily guarantee improved catalytic stability. In fact, stability may even decrease over time due to potential aggregation or accumulation between catalyst particles at high loadings. This aggregation creates localized regions of high current density, leading to accelerated catalyst degradation, including increased corrosion or surface passivation. By understanding the factors influencing catalyst deactivation and the reasons behind it, new strategies can be formulated to design catalysts with enhanced stability, thus aligning with the demands of practical applications.

### 3.2 Physio‐Chemical Characterization Methods

While electrochemical tests are fundamental in evaluating HER catalysts, they alone do not provide a comprehensive understanding. Supplementary characterization techniques are essential for a deeper understanding of catalyst structure, surface‐interface properties, product distribution, etc. These techniques help explain the observed trends in activity and stability during electrochemical measurements (**Table** **2**). Currently, ex situ techniques are commonly used to show the state of catalytic materials before and after reactions, which is crucial in determining changes in catalytic performance and the location of active sites. However, ex situ techniques often fail short in providing a complete and accurate understanding of the reaction mechanism. The advancement of in situ characterization techniques not only sheds light on the real‐time reaction process but also offers preliminary insights into catalyst pre‐treatment, surface/structure reconstruction processes, and the identification of genuine active sites within the catalyst.^[^ ^57^ ^]^ Additionally, in situ characterization can provide reliable visual evidence of physical or chemical changes in the electrocatalyst structure and local electrolyte.^[^ ^58^ ^]^

**Table 2:** Physio‐chemical characterization methods for HER catalysts.

	Characterization technology	Parameters extracted	Advantage	Disadvantage
Ex situ	Scanning electron microscopy (SEM)	Microscopic surface morphology	Large sample observation capability. Rapid imaging	Can't provide chemical information. Need for conductive coating
	Energy‐dispersive X‐ray (EDX)	Element distribution	High spatial resolution. Non‐destructive. Rapid analysis.	Difficulty detecting low‐energy elements. Elemental characterization rather than quantification. 3) Overlapping peaks and spectral interference.
	Transmitting electron microscopy (TEM)	Microstructure	Atomic level. Thickness measurement. Microscopic area selection.	Complicated sample preparation. Limited to nanoscale. Destructive observation.
	High‐angle annular dark‐field scanning TEM (HAADF‐STEM)	Atomic structure, Morphology at the nanoscale	High‐resolution imaging. Atomic resolution imaging. 3D reconstruction capability.	High sample preparation requirements. Complexity of instrumentation.
	Atomic force microscopy (AFM)	Surface mechanical properties	Multiple measurement modes. sub‐nanometer scale surface topography images.	Limited scanning speed. Surface interaction effects.
	X‐ray diffraction (XRD)	Chemical composition, Phase	Quantitative analysis. Non‐destructive analysis	Unable to observe amorphous materials. High sample preparation requirements.
	X‐ray photoelectron spectroscopy (XPS)	Chemical composition, Oxidation states	Surface sensitivity. Non‐destructive. Quantitative analysis	Limited internal structure. Limited depth of information. High sample surface requirements.
	Fourier transform infrared spectroscopy (FTIR)	Adsorbed species, reaction intermediates	Non‐destructive analysis. Fast response. Quantitative analysis.	Limited surface sensitivity. Complex interpretation. Susceptible to interference from the ambient atmosphere.
	Brunauer–Emmett–Teller (BET)	Porosity analysis, Specific surface area	Good repeatability. High sensitivity. Non‐destructive.	Assumption limitations. Single adsorbed molecule limitation. It does not provide detailed information about pore structure and size distribution.
In situ	In situ X‐ray absorption spectroscopy	Charge transfer and ion diffusion processes, Edge structure, Transition state	Real‐time observation. Elemental selectivity. High energy resolution. Fusion of structural and electronic information	Requires a high vacuum or inert gas environment. Limited by catalyst transparency. Complicated data processing and interpretation.
	In situ Raman spectroscopy	Structural transition, Interfacial species on the surface,	Provided molecular characterization spectroscopy in real‐time. Non‐invasive. High sensitivity.	Inability to detect non‐polar species or certain metal ions. Complex data interpretation. Low signal on inactive catalyst surfaces.
	In situ TEM techniques	Material growth, Transformation processes	Multiple imaging and analysis modes. Multi‐scale observation. High spatial resolution	Demanding experimental conditions. Sample limitation. Affected by beam irradiation.
	In situ infrared spectroscopy	Molecular adsorption, Reaction product formation processes	Flexible experimental conditions Non‐invasive. Real‐time monitoring of changes in chemical characterization information in the reaction	Complexity of species identification. Weak detection of deeply adsorbed or internal species
	In situ mass spectrometry	Generation and disappearance of substances	Multi‐component analysis. Detect species at low concentrations.	Reaction condition limitations. Complexity of data interpretation

## 4 Material Engineering Strategies for High‐Performance HER Catalysts

Noble metal‐free HER catalysts are usually classified by composition, including metal sulfides,^[^ ^59^, ^60^ ^]^ metal selenides,^[^ ^61^, ^62^, ^63^ ^]^ metal carbides,^[^ ^64^, ^65^ ^]^ metal nitrides,^[^ ^66^, ^67^ ^]^ metal phosphides^[^ ^68^, ^69^ ^]^ and metal‐free catalysts.^[^ ^70^, ^71^ ^]^ Transition metals (Mo, W, Co, Ni, Fe, etc.) are considered promising substitutes for noble metals as HER catalysts and are commonly used in laboratory investigations of noble metal‐free catalysts.^[^ ^72^ ^]^ Conventional noble metal‐free catalysts exhibit a certain gap in performance compared to noble metal catalysts. To achieve the goal of showcasing HER catalytic performance comparable to noble metals at a low cost, it is necessary to modify noble metal‐free catalysts using different design strategies. The strategies discussed in this section center on optimizing the performance of noble metal‐free catalysts through material engineering approaches, considering their unique characteristics. The primary objectives of these strategies are to: 1) enhance the density of active sites; 2) reduce interfacial transfer resistance and improve electronic conductivity, facilitating more efficient electron transfer during the reaction; 3) enhance reaction kinetics and lower hydrogen adsorption Gibbs free energy, making the process more energetically favorable and 4) improve catalyst stability while maintaining catalytic activity, striking a balance between durability and performance.

### 4.1 Doping

**Figure 4:**
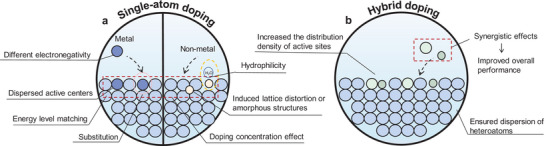
Schematic representation of doping strategies to improve catalytic performance. The catalysts are modified by the unique properties of the dopant atoms to achieve improved performance, which includes a) single‐atom doping and b) hybrid doping.

Atom‐scale doping is an effective strategy for enhancing the activity of catalysts. **Figure** **4** illustrates various types of doping approaches, with labels highlighting their effects on the catalyst. Commonly, single‐atom doping involves metallic and non‐metallic atoms, as well as heteroatoms, to modify HER catalysts.^[^ ^73^, ^74^ ^]^ The main goal of doping is to alter the coordination environment and electron density of the catalyst through the unique properties of the dopant atoms. Such adjustments optimize adsorption and desorption energies of reactants, consequently enhancing catalytic activity. In addition, doping process leads to partial substitution of catalyst structure, which alters the electronic structure and thus makes it more conductive for charge carriers diffusing within the electrochemical double layer so as to improve adsorption at the catalytic surface.^[^ ^75^ ^]^

Dopant atoms interact with the orbitals of catalysts, leading to a downward shift of the d‐band center and a reduction in the Gibbs free energy of hydrogen adsorption.^[^ ^76^ ^]^ This substitution optimizes the local coordination environment, which can significantly influence catalytic activity when various coordination bonds are present.^[^ ^77^ ^]^ Different metal dopants have distinct effects on the catalyst. For instance, the doping of highly positively charged metal element, e.g., Mo, promotes the nucleophilic adsorption of water, enhancing the surface charge transfer.^[^ ^78^ ^]^ In alkaline conditions, Fe doping improves the ability of catalysts to dissociate water during the reaction.^[^ ^79^ ^]^ Metal elements that serve as active sites increase the number of such sites within the catalyst, thereby boosting catalytic efficiency. Moreover, non‐metal doping is also highly effective in enhancing HER activity. Non‐metal doping modulates the charge transfer, increases conductivity and employs affordable and readily available elements, reducing the reliance on rare metals and the overall cost of catalyst preparation. However, single‐atom doping using non‐metal atoms can produce a redistribution of charge that may further induce lattice distortions or amorphous structures.^[^ ^80^ ^]^ Although it can increase the number of active sites, e.g., S,^[^ ^81^ ^]^ these sites are relatively limited and may be less advantageous compared to metal single‐atom doping in terms of catalytic activity.

Hybrid doping, which combines different elements, emerges as a promising approach in HER catalysis, often outperforming monoatomic doping (Figure 4b). This method leverages the synergistic effect to enhance both catalytic activity and stability of the HER.^[^ ^82^ ^]^ Hybrid doping delivers multifunctionality upon catalysts, as various heteroatoms can create different types of active sites on the surface, each with unique catalytic activities and selectivities.^[^ ^83^ ^]^ Introducing heteroatoms can also modify the lattice structure and chemical bond strength of the catalysts, and meticulous modulation of these aspects can enhance the structural stability.^[^ ^68^ ^]^

The doping strategy proves effective in enhancing catalyst activity with a minimal number of atoms, making it a cost‐effective approach. In doping engineering, precise control over the placement and concentration of dopant elements is crucial. These elements need to be positioned strategically to interact synergistically with the active metal, avoiding adverse interactions that could affect the structural stability and slow down the reaction. In particular, the concentrations of dopant atoms play a significant role in determining the catalyst activity, thus requiring a well‐balanced approach to ensure both catalytic performance and structural stability.

### 4.2 Vacancy Defect

The creation of vacancy defects serves dual purposes for enhancing HER activity. First, it exposes more active sites and enhances the utilization of active sites within. Second, these defects facilitate fast charge transfer and increase conductivity. Currently, two main methods are employed to create vacancy defects, introducing atomic vacancies and exposing active site edges.

**Figure 5:**
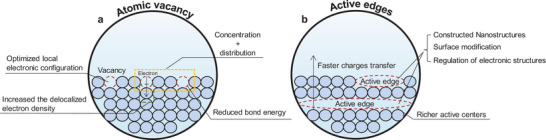
Schematic representation of vacancy strategies to improve catalytic performance. Vacancy defects improve the utilization of active sites while being able to increase the conductivity, where the vacancy strategy consists of a) atomic vacancy and b) exposure of active edges.

The introduction of vacancies in a catalyst, characterized by the absence of atoms at specific lattice sites, is a refined method to adjust the electronic structures of catalysts. Single‐atom vacancies, in particular, are effective in this regard (**Figure** **5**a). The formation of vacancies leads to decreased bonding energy, as the localized electronic states of the vacancies hybridize with hydrogen electronic states, forming bonding states. Additionally, the energy required for vacancy formation converts into an increased hydrogen adsorption energy. This process promotes a preferential flow of residual electrons around other metal atoms, creating an electron‐rich region that enhances hydrogen adsorption.^[^ ^84^ ^]^ The vacancy‐based strategy enables the attainment of optimal catalysts by modulating the content and surface morphology of vacancies.^[^ ^85^ ^]^ However, similar to the doping strategies, it is also crucial to elegantly control the vacancy concentration, as excessive vacancy generation can detrimentally impact electrocatalytic performance and potentially damage the crystal strcutre.^[^ ^86^ ^]^

The creation of active edges is another vacancy‐creating approach for enhancing the HER activity (Figure 5b). To effectively construct the active edges, there are three main approaches.^[^ ^87^ ^]^ 1) Utilizing nanostructures. These structures include nanoparticles, vertical nanocrystals, mesoporous double gyroscopic structures, and molecular clusters. 2) Introducing additional active components by utilizing the active edges. 3) Fine‐tuning the electronic structures. Active edges offer a higher concentration of unsaturated bonds, leading to enhanced reactivity. DFT studies have demonstrated that the Gibbs free energy of hydrogen adsorption on active edges is close to zero, distinguishing them from other active sites.^[^ ^88^ ^]^

Vacancy defect currently serves as an effective design strategy for enhancing the catalytic performance of HER. The catalytic performance is directly influenced by the atomic radii and electronegativity differences among various elements. Hence, investigating the catalytic process mechanism of vacancy defects induced by different elements and exploring the synergistic effects between different vacancies are crucial directions for future development. Moreover, it is essential to dedicate attention to studying the dynamic evolution of vacancies during the catalytic process to gain a deeper understanding of the catalyst activity's origin.

### 4.3 Phase Engineering

**Figure 6:**
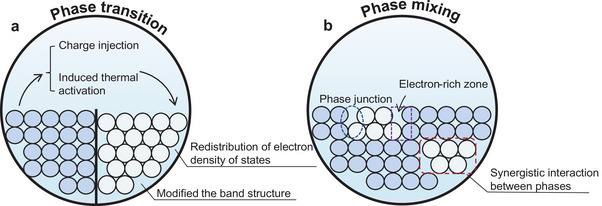
Schematic representation of phase engineering affecting catalytic performance. a) Phase transitions of catalysts can reconfigure the electronic state density and modify the energy band structure. b) Localized generation of phase mixing enables synergistic effects of different phases.

Phase engineering plays a crucial role in constructing high‐performance catalysts, as the crystal phases are closely linked the intrinsic properties of catalysts, such as d‐band position, conductivity, structure and chemical stability.^[^ ^89^ ^]^ Phase transitions induce changes in electronic configuration, splitting of electronic energy levels, and modifications in ligand environments, thereby influencing the activity of catalyst and selectivity (**Figure** **6**a).^[^ ^90^ ^]^ Integrating electrocatalytic activity with phase structure offers valuable insights into catalyst design principles. The methods employed to induce phase transitions involve **charge injection** and **induced thermal activation**. The former can be achieved by chemical stripping with insertion of Li or Na ions,^[^ ^91^ ^]^ and the latter involves strain fields and high‐energy ion injection to induce phase transitions.^[^ ^92^ ^]^ MoS_2_ is the most typical transition metal sulphides compound used in crystalline phase transformations, including 1T, 2H, and 3R phases. Crystal field theory provides an explanation for the phase transition of transition metal‐based catalysts.^[^ ^93^ ^]^ The rate of phase transition is determined by the energy barrier between the two phases, with a lower energy barrier corresponding to a faster phase transition process.

In addition, the activity of catalysts can be further optimized through interfacial synergy by utilizing mixed phases generated via local phase transitions (Figure 6b). The ensemble effect of synergy between two or more structures on the Faraday electron transfer process can be induced through the accumulation of surface active sites, interfacial charge transfer and modulation of the electronic structure.^[^ ^94^ ^]^ By exploiting the energy level differences between crystalline phases, heterogeneous junctions can be formed at interfaces, resulting in a directional transfer of electrons. This generates electron‐enriched regions in the interface of the phase‐junctions, thereby optimizing the electronic structure of the catalyst.^[^ ^95^ ^]^ Furthermore, phase mixing can enhance the covalent bonding between atoms, capable of improving the robustness of the lattice and enhancing the stability of the catalysts.^[^ ^96^ ^]^ By employing these strategies, it is possible to construct mixed‐phase catalysts in catalytic materials with various crystalline phases and regulate their mixing ratios to further enhance the HER activity.

Phase engineering is a commonly used strategy for HER catalyst design, but the investigation of amorphous phases should not be overlooked. The amorphous phase holds significant potential due to its abundant active sites and flexible structure, necessitating exploration of controllable synthesis strategies. Currently, phase regulation is still synthesized using a trial‐and‐error method. More efficient methods are needed to achieve precise regulation.

### 4.4 Strain Engineering

Strain engineering, a technique to modify strain, is highly effective in tailoring the properties of catalysts. It involves the application of tensile or compressive strains which result from lattice expansion or contraction, as well as strains induced by substrates. By manipulating the lattice constants, strain engineering can alter the intrinsic atomic spacing, modify the energy levels of the bonding electrons, significantly reduce the energy barrier for the HER, and thus optimize the HER dynamics.^[^ ^97^ ^]^

**Figure 7:**
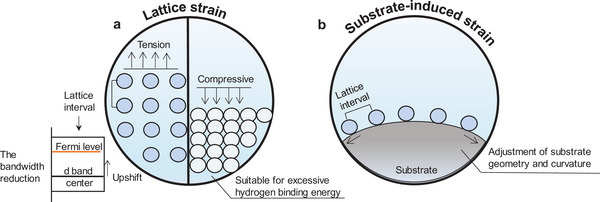
Schematic representation of the effects of strain engineering on catalytic reactions. a) Effect of lattice strain generation, which includes tension and compressive stresses. b) Strain engineering resulting from substrate induction.

When tensile strain is applied, atomic spacing increases, causing a decrease in d‐band electron overlap between adjacent atoms (**Figure** **7**a). This reduction narrows the bandwidth and shifts the center of the d‐band closer to the Fermi energy level, enhancing the interaction force between the metal and hydrogen atoms. Conversely, compressive strains shift the center of the d‐band downwards, weakening the metal‐H interaction and reducing the H adsorption energy. Compressive strain is particularly suitable for materials with strong H‐binding energy, as it further decreases the H adsorption energy, making it more favorable for H_2_ precipitation. This strategic manipulation of strain opens up new possibilities for designing and optimizing catalysts for the HER. Furthermore, the induction of the substrate can also result in strain effects on the catalyst bound to it. The impact of substrate‐induced strain largely relies on the geometrical distribution and curvature of the substrate, which in turn reduces the internal resistance to charge transfer and thereby enhancing the performance.^[^ ^98^ ^]^

It is important to note that strain engineering is often implemented alongside other strategies, making it challenging to isolate its specific effects. Incorporating strain engineering as a supplementary enhancement strategy to improve catalytic performance in conjunction with other strategies is an effective design tool. Despite the acknowledged benefits of strain engineering, a gap remains in our understanding, primarily due to the lack of studies focusing exclusively on property changes induced by strain effects and revealing the exact mechanisms relevant to strain engineering. Therefore, further investigation into the sole impact of strain engineering is crucial for a more accurate and comprehensive understanding of this strategy in catalyst optimization.

### 4.5 Heterogeneous Interface

**Figure 8:**
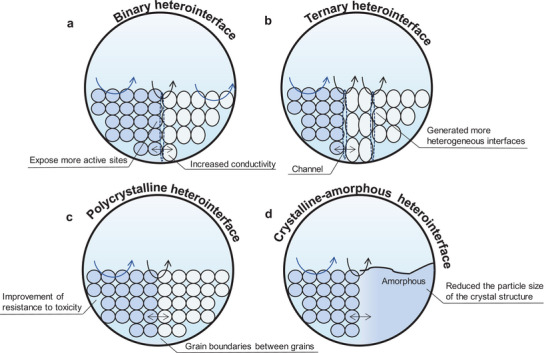
Schematic representation of the role of four different heterogeneous interfacial structures, including a) binary heterointerface structure, b) ternary heterogeneous structure, c) polycrystalline heterointerface structure and d) crystalline‐amorphous heterointerface structure.

Heterogeneous interfacial structures typically form between two or more distinct components. **Figure** **8** summarizes the effect of generating different heterogeneous interfaces on catalytic reactions. Creating heterogeneous interfacial structures can tune the electronic properties and adsorption behavior of the catalyst so as to influence the reactivity of the reactants on the catalyst.^[^ ^2^ ^]^ Strong coupling interactions resulting from heterogeneous interfaces can also enhance the durability of the electrocatalytic process. Generally, heterogeneous interfaces give rise to two distinct reaction pathways:^[^ ^99^ ^]^ I) the first involves the heterogeneous interface serving as a channel for electron transfer, with the reaction primarily occurring on one of the components; II) the second involves the heterogeneous interface acting as a channel for providing reaction intermediates, leading to the adsorption and desorption processes of the different components, respectively.

Heterogeneous interfaces integrate active centers from different moieties, inducing electron redistribution around the reaction centers and concentrating a significant number of central electrons at the interface. This results in the generation of additional active sites, the formation of multi‐site reaction pathways, and a reduction in the energy barrier for electrochemical reactions.^[^ ^100^ ^]^ The majority of heterogeneous structures involve the regulation of two distinct components, namely binary heterointerface (Figure 8a). Furthermore, ternary heterogeneous structures have also garnered attention (Figure 8b).^[^ ^101^ ^]^ In comparison to binary heterogeneous structures, ternary structures exhibit more intricate interface structures and yield a higher number of active sites. Additionally, the introduction of a third component can augment the adsorption sites on the catalytic reaction surface, diminish the coverage of toxic substances, thereby improving the durability of the catalyst. Nevertheless, it is crucial to consider the complexity associated with synthesizing ternary catalysts and the limitations concerning to more intricate performance optimization.

Given the complexity and uncertainty in creating desired heterogeneous interfaces between various components, selecting materials with compatible crystal structures and properties is crucial. In comparison to binary heterogeneous interfacial structures, polycrystalline types offer a larger specific surface area and an increased number of reactive sites for catalytic reactions (Figure 8c).^[^ ^102^ ^]^ Polycrystalline heterostructures exhibit grain boundaries between the grains, which can impede grain diffusion and growth. These grain boundaries also act as barriers to the diffusion and adsorption of toxicants, thus enhancing the catalytic durability and stability of the catalyst.^[^ ^103^ ^]^ Therefore, the influence of factors such as grain boundaries and crystal structure inhomogeneity must be considered during the design and synthesis process. While the construction of polycrystalline heterointerfaces is relatively straightforward, it is necessary to account for the complex effects of grain boundaries and the regulation of crystal orientation. Additionally, conventional characterization methods tend to provide an overview of the structure, necessitating more precise characterization techniques to analyze the details of the grain boundary regions. Furthermore, the use of heterogeneous structures resulting from crystal‐amorphous interactions can optimize catalyst activity (Figure 8d).^[^ ^104^ ^]^ This is attributed to the introduction of an amorphous phase, which reduces the particle size of the crystal structure and thereby increases both activity and durability. Achieve precise control and modulation of heterogeneous interfacial structures remain a significant challenge in the field, which necessitates the development of refined synthetic methods and techniques to precisely control the atomic arrangement, lattice matching and chemical composition of interfaces.

Heterogeneous interfaces as a common design strategy for non‐precious metal catalysts is crucial for improving catalytic activity. However, few studies have been able to scientifically explain the process of complex heterointerfaces, which hinders the further improvement of constructed heterointerface catalysts. In the future, it may be possible to develop more reliable and controllable growth and assembly techniques to achieve precise control and assembly of heterogeneous interfaces, which could potentially modulate catalytic performance.

### 4.6 Metal‐Support Interaction

The performance of catalysts is significantly influenced by the physical and chemical interactions between active components and carriers. Support plays a dual role in catalysts: first, it facilitates the dispersion of nanoparticles, thereby increasing the utilization of catalytic active sites; second, it serves as a medium for electron transport and enables the diffusion of the catalyst.

**Figure 9:**
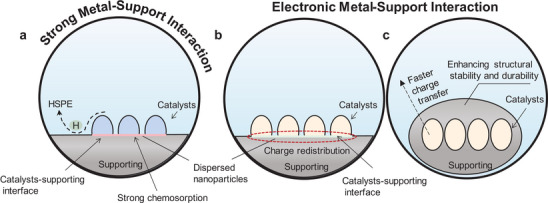
Schematic representation of metal‐support interaction. Support can facilitate the dispersion of catalytic nanoparticles as well as increase the conductivity. There are two main forms of a) strong metal‐support interactions and electronic metal‐support interactions, where the electronic metal‐support interactions are in the form of b) substrate and c) coating structures.

The strong metal support interaction (SMSI) is the prototypical metal‐support interaction (**Figure** **9**a).^[^ ^105^ ^]^ In SMSI, the metal is dispersed on the surface of the carrier, allowing for efficient charge transfer from the surface of the metal‐based catalyst to the support. This process is known as the hydrogen spillover effect (HSPE). The action of the HSPE overcomes the free energy of hydrogen adsorption and facilitates the desorption of H as a molecule.^[^ ^106^ ^]^ The strong chemisorption of metals on the support hinders their agglomeration and enhances atom utilization, thereby facilitating effective and long‐lasting catalysis of the HER.

Another type of metal‐support interaction is electronic metal support interaction (EMSI). EMSI refers to the redistribution of charge at the interface between loaded metal nanoparticle and the support. The interaction and electron transfer between the metal and the support can alter the electronic state and electron density of the metal surface. The support can modify the electronic structure of the metal surface through electron donation or acceptance. Commonly used supports include carbon‐based materials (such as graphene oxide,^[^ ^107^ ^]^ porous carbon,^[^ ^108^ ^]^ and N‐doped carbon),^[^ ^109^ ^]^ MOF,^[^ ^110^ ^]^ and MXene.^[^ ^8^ ^]^ It can be present as substrates or coatings. As a substrate, they form an electron cloud that can modulate the adsorption capacity of reactants on the metal surface and the configuration of reactants at the active site (Figure 9b). Simultaneously, the energy barrier of the reaction process is lowered, facilitating the dissociation of H_2_O and desorption of H_2_.^[111]^ Coating, carriers provide a larger specific surface area, mitigating the adverse effects of monolayer aggregation(Figure 9c).^[^ ^112^ ^]^ Additionally, interfacial interactions enhance charge transfer ability and exhibit superior electrochemical performance.^[^ ^113^ ^]^ Moreover, the use of cladding, a special structure, can impede migration from the catalyst surface and ensure structural stability and activity durability.

Metal‐support interaction pose several challenges in catalysts. First, these interactions occur at the interface, necessitating compatibility and interfacial matching between the metal and the support. Factors such as lattice matching and differences in thermal expansion coefficients among materials can result in interfacial stress and instability, potentially impacting catalyst performance. Additionally, it can induce structural alterations and surface remodeling of the catalyst, which may influence its activity, selectivity, and lead to catalyst deactivation.

### 4.7 Current Situations Using Noble Metal‐Free Catalysts

The noble metal‐free catalysts prepared based on the above strategy are intended to be applied to the technology of industrial hydrogen production from electrolyzed water to achieve cost reduction and efficiency improvement.^[^ ^114^, ^115^, ^116^, ^117^ ^]^ Currently, there are four primary technologies for hydrogen production through water electrolysis:^[^ ^118^ ^]^ alkaline water electrolysis (AWE), proton exchange membrane electrolysis (PEM), anion exchange membrane electrolysis (AEM), and solid oxide electrolysis (SOE). **Table** **3** provides the basic parameters and characteristics of these four water electrolysis technologies.

**Table 3:** Basic information on the four major electrolytic water‐to‐hydrogen technologies, including electrolytes, operating environments, advantages, limitations, and levels of technological maturity.

Technology	Alkaline water electrolysis (AWE)	Proton exchange membrane electrolysis (PEM)	Anion exchange membrane electrolysis (AEM)	Solid oxide electrolysis (SOE)
Electrolyte	30‐40 wt.% KOH	H_2_SO_4_/HClO_4_	KOH/Pure water	Y_2_O_3_/Zr_2_O_3_
Operating condition	70‐90 °C 1–30 bar	50‐80 °C 15–30 bar	50‐85 °C 15–30 bar	700‐1000 °C < 30 bar
Advantage	Available inexpensive noble metal‐free catalysts. Low cost. Rapid start/stop.	High dynamic response. High hydrogen evolution purity. High ionic conductivity and current density	Stable operation. Low cost. Feasibility of using noble metal‐free catalysts.	High conversion efficiency. Decrease the voltage. 3. Increase the rate of reaction.
Limitations	Dealkalization required after gas production. Low hydrogen production rates. Sensitivity to differential pressures	Poor electrolyzer life. High capital cost. High platinum group metal loading required for the electrodes.	Slow dynamic response. Low conductivity. Poor alkali resistance.	High ohmic loss. Poor durability. 3. Inconvenient start/stop.
Technology readiness level	Mature	Preliminary	Early development stage	

Nevertheless, hydrogen production using laboratory‐prepared noble metal‐free catalysts typically occurs at low current densities (1–100 mA cm^−2^). It is imperative to investigate non‐precious metal catalysts at high current densities (>1000 mA cm^−2^) on an industrial scale.^[^ ^119^ ^]^ Limited documentation exists regarding noble metal‐free catalysts operating at high current densities, but notable advancements have been made. The utilization of a hetero‐interfacial strategy in Ni(OH)_x_/Ni_3_S_2_ catalysts has facilitated strong electronic coupling, enabling them to achieve a current density of 1000 mA cm^−2^ with 238 mV overpotential.^[^ ^120^ ^]^ Moreover, NNAs catalysts prepared on nickel foam using electrodeposition were grown with non‐equilibrium helical dislocations. Due to the special crystalline and morphological characteristics, NNAs require a low overpotential of 469 mV at 5000 mA cm^−2^ current density in alkaline HER, which has a great potential for application in AEW.^[^ ^121^ ^]^ In the presence of high current densities, it is crucial to consider the impact of gas bubble on catalytic performance. The rapid formation of numerous bubbles can aggregate at the interface between the catalyst and electrolyte, hindering the reaction at the active sites of the catalyst and thus impairing its performance.^[^ ^122^ ^]^

## 5 Summary and Outlook

**Figure 10:**
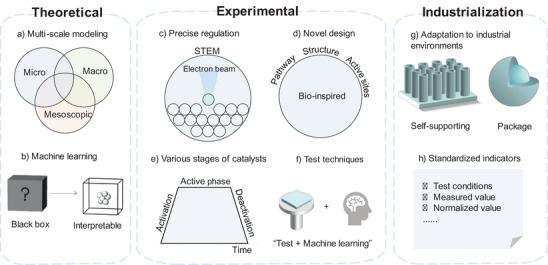
Future perspectives on noble metal‐free HER catalysts in three directions: theoretical, experimental, and industrialization. a) Theoretical computational studies of HER at multiple scales. b) Realizing the interpretability of machine learning models. c) Improving the precision control of catalyst preparation. d) Preparation and design of new catalysts with improved activity and stability. e) Study of catalyst processes at different stages. f) Establish more accurate characterization techniques by combining characterization methods with ML. g) Preparation of catalysts that can be adapted to industrial production environments, such as self‐supported structures and package structures. h) Setting of catalyst performance specifications for industrial production.

It is true that the performance of current state‐of‐the‐art noble metal‐free catalysts still falls short of the requirements for practical applications. **Figure** **10** provides a summary of various research directions that noble metal‐free catalysts can pursue in the future, considering the theoretical foundations, experimental investigations and industrial applications.

### 5.1 Development of Interpretable High‐Precision Theoretical Models

The study of the origin of hydrogen precipitation activity of different catalysts is presently mainly based on DFT for atomic scale simulation, and the main computational programs are mainly VASP, CP2K and Quantum‐Espresso.^[^ ^123^, ^124^ ^]^ Previous research on catalytic mechanisms lacks investigation into complex surface interfaces and actual reaction situations. There is an immediate necessity for more precise models to explore the intrinsic nature of catalysis at the atomic scale, enabling better comprehension of the mechanisms behind HER, the dynamic alterations of active sites, and the ability to predict more suitable materials. Scientific methods that incorporate multiple scales, such as integrating quantum mechanics calculations, molecular dynamics simulations, and continuum models, have the capability to simulate the formation and reaction processes occurring on catalyst surfaces at the active sites. These methods can be combined with macroscopic reaction kinetics to achieve comprehensive multi‐scale modeling and computation (Figure 10a). Another potential direction for the advancement of multi‐scale computational modeling methods is the coupling of ab initio constrained thermodynamic and microscopic dynamic models.

Machine learning (ML) is an essential tool for data mining and analysis, which enhances the conventional approach to discovering novel catalysts by leveraging accumulated experience and significantly reducing the costs associated with trial and error.^[^ ^125^ ^]^ A critical step in the machine learning process is data collection. Electrochemical catalysts commonly rely on three primary sources of data: experimental data, computational and simulation data, and large databases.^[^ ^126^ ^]^ Some databases contain the basic properties of the underlying material, including descriptors, crystallographic properties of the material, and physicochemical properties, avoiding the expensive costs associated with self‐built databases. To guarantee the quality of the machine learning model, it is crucial to thoroughly assess the collected data for completeness, consistency, accuracy, and validity.^[^ ^127^, ^128^ ^]^ ML possesses the unique ability to identify hidden patterns or correlations in high‐dimensional data, enabling the exploration of non‐linear relationships and implicit rules in catalyst activity and stability for the HER (Figure 10b).^[^ ^129^, ^130^ ^]^ While commonly used black‐box models can predict descriptors such as adsorption or formation energies, interpreting the internal logic of these complex models proves challenging. Interpretable machine learning methods integrate the predictive power of black‐box models with the physical interpretability of physics‐based models to improve the prediction of real catalytic materials' behavior under reaction conditions.^[^ ^131^ ^]^ However, these computational tools typically require substantial amounts of data for training and optimizing behavioral patterns. Meta‐learning is a promising solution for small datasets. It can learn relationships from correlation models trained on large datasets and apply this prior knowledge to small datasets. Similarly, developing reverse design models, like STONED,^[^ ^132^ ^]^ enables model iteration and optimization with reduced data demands when combined with active learning techniques.

### 5.2 Optimization of Catalyst Design and Preparation

It is worth noting that there are still challenges in how to precisely control catalysts based on design strategies in order to fully exploit their catalytic performance. The use of electron beam‐induced precise atomic doping and multi‐atom assembly in scanning transmission electron microscopy (STEM) holds the potential to combine machine learning automated data processing and image feedback to achieve the vision of atomically precise manufacturing (Figure 10c).^[^ ^133^ ^]^ Biomimetic catalysis design, inspired by natural concepts, holds promising prospects for development. For instance, investigating the metabolic pathways of hydrogen‐producing microorganisms like microalgae and incorporating their principles into catalyst production design may lead to unforeseen outcomes (Figure 10d).^[^ ^134^ ^]^ Enzyme catalysis, known for its remarkable reaction kinetics in nature, provides an opportunity to explore enzyme structure, elements, and reaction mechanisms for novel noble metal‐free catalyst design. Drawing inspiration from the structure and active sites of [Fe–Fe] hydrogenase, novel iron‐based bifunctional catalysts can be synthesized to efficiently transfer H_2_ and protons under near‐neutral conditions.^[^ ^135^ ^]^ Furthermore, the catalytic promiscuity observed in enzymes may offer insights for the design and preparation of dual‐functional noble metal‐free catalysts. In biological enzymes, residues near the active center play a crucial role in regulating enzyme activity. In polymers, by utilizing Co‐porphyrin as the reaction center and incorporating three different side‐chain groups to simulate the residues controlling activity in biological enzymes, these side‐chain groups can regulate the hydrogen evolution activity of the catalyst.^[^ ^136^ ^]^ The structures found in nature can transcend their own formation methodology. Covalent organic frameworks (COFs) based electro‐catalysts have the ability to precisely manipulate pore structures and functional groups through chemical manipulation. This facilitates the accurate introduction of active sites into long‐range ordered channels and accessible surfaces of COFs.^[^ ^137^ ^]^

### 5.3 Comprehensively Understand the Various Stages of Catalysts

While considering catalyst performance, it is vital not to overlook the long‐term stability of catalysts. Most catalyst research literature focuses solely on the three steps of “design‐preparation‐testing”. However, a more comprehensive approach is needed, taking into account each stage of catalysis, including activation, active phase, and deactivation, and conducting an analysis of the causes of deactivation (Figure 10e). The activation stage reflects the initial rate of hydrogen evolution, and a prolonged activation time indicates lower efficiency in the initial reaction stage, thereby reducing the overall efficiency of the reaction process. The deactivation mechanism of hydrogen evolution electrocatalysts is a complex issue, encompassing oxide formation, gas bubble formation, corrosion and dissolution, accumulation of reaction intermediates, and loss of active sites.^[^ ^54^ ^]^ Investigating the factors leading to catalyst deactivation can serve as a reference for the early‐stage design of catalysts.^[^ ^138^ ^]^

### 5.4 Next‐Generation Testing Protocols and Techniques

Exploring the structure‐composition‐performance relationship at the atomic level necessitates employing various advanced in situ characterization techniques to monitor the operational structural evolution of catalysts. Current in situ testing techniques are intricate and expensive in terms of testing and analysis procedures. The incomplete dynamic tracking of physicochemical properties on electrocatalyst surfaces under reaction conditions, coupled with the insufficient provision of detailed information regarding catalyst surface evolution, underscores the need to enhance the sensitivity, response time, and data acquisition speed of in situ characterization methods. Furthermore, precision in catalyst measurements warrants improvement. For instance, a quartz microbalance can detect mass changes at the nanogram‐level, thereby offering a more accurate reflection of mass variations pre‐ and post‐catalytic reactions. Additionally, emphasis should be placed on investigating the interfacial properties of catalysts. In situ liquid‐phase 4D ultrafast transmission electron microscopy, employing a single‐pulse electron imaging mode, facilitates observation within nanosecond electron detection pulses, precisely controlling time delay, thus enabling the study of nanoscale kinetic processes at the gas‐liquid interface.^[^ ^139^ ^]^ The combination of “characterization techniques + ML” can enhance testing speed and automatically interpret complex data (Figure 10f). Examples include “XAS + neural network”, “Raman + AI”, and “XRD + convolutional neural network”.^[^ ^129^, ^140^, ^141^ ^]^ The rapid progress of machine learning is anticipated to enhance the sensitivity of low‐dose testing in the testing process and attain real‐time feedback.

### 5.5 Bridging the Gap Between Experimental and Industrial Applications

Industrial production is conducted at a high current density, and efficient hydrogen evolution necessitates significant charge transfer and rapid reactions at active sites. Mechanical stirring, elevated reaction temperatures, and high electrolyte concentrations are commonly employed in industry to enhance catalytic efficiency, which requires catalysts with greater stability. Currently, the widely used Pt/C catalyst in industry possesses an electrochemically active surface area >65 m^2^ g^−1^ and particle size of ≈2–4 nm. In laboratory tests utilizing a three‐electrode device (1 m KOH, current density of 10 mA cm^−2^), the overpotential of the commercial 20 wt.% Pt/C catalyst is ≈38 mV, with a Tafel slope ranging from 29–31 mV dec^−1^.^[^ ^115^ ^]^ In contrast, noble metal‐free HER catalysts prepared by researchers exhibit an overpotential of ≈35–150 mV and a Tafel slope exceeding 35 mV dec^−1^.^[^ ^142^ ^]^ While noble metal‐free catalysts have demonstrated competitive performance compared to commercial catalysts at low current densities, there remains substantial room for improvement at high current densities. Self‐supported catalysts have exhibited exceptional performance at high current densities as they are in situ grown on conductive substrates, ensuring electrical conductivity while minimizing binder usage (Figure 10g). For instance, self‐supported porous cobalt phosphide (Co–P) foam has demonstrated remarkable stability in alkaline environments at a current density of 1000 mA cm^−2^ for 3000 h without significant degradation.^[^ ^142^ ^]^ To achieve catalysts with even superior stability, metal particles can be encapsulated in mesoporous silica, zeolites, or core‐shell structures to extend the lifetime of active components, mitigate rapid corrosion and structural collapse caused by high‐concentration electrolytes during hydrogen evolution. The performance indicators and evaluations obtained in laboratory settings are inadequate for assessing suitability on an industrial scale; therefore, it is imperative to establish catalyst performance evaluation standards relevant to industrial applications (Figure 10h).^[^ ^143^ ^]^ For example, R_η/j_ which quantifies the required overpotential as the current increases, can serve as an indicator for evaluating catalyst performance at high current densities.^[^ ^144^ ^]^ When addressing large‐sized electrodes, performance evaluation standards for industrial applications should also consider factors such as mass transfer efficiency, electric field distribution, and other relevant considerations. We believe our perspective article will provide practical solutions transitioning the experimental outcomes^[^ ^145^, ^146^, ^147^ ^]^ to real‐world applications.

## Conflict of Interest

The authors declare no conflict of interest.
